# Supplementary material for: Dung removal increases under higher dung beetle functional diversity regardless of grazing intensification
Source: Nat Commun. 2023 Dec 6;14:8070. doi: 10.1038/s41467-023-43760-8 (PMC10700315; doi:10.1038/s41467-023-43760-8)
Supplement: Supplementary file 1 — Supplementary Information [file 41467_2023_43760_MOESM1_ESM.pdf]

Supplementary Information for:

Dung removal increases under higher dung beetle functional diversity regardless of grazing intensification

Jorge Ari Noriega[1,2,†], Joaquín Hortal[1,3,4;*,†], Indradatta deCastro-Arrazola[1,5], Fernanda Alves-Martins[1,6,7], Jean C.G. Ortega[3,8], Luis Mauricio Bini[3], Nigel R. Andrew[9], Lucrecia Arellano[10], Sarah Beynon[11], Adrian L.V. Davis[12], Mario E. Favila[10], Kevin D. Floate[13], Finbarr G. Horgan[14,15], Rosa Menéndez[16], Tanja Milotic[17], Beatrice Nervo[18], Claudia Palestrini[18], Antonio Rolando[18], Clarke H. Scholtz[12], Yakup Senyüz[19], Thomas Wassmer[20], Réka Ádam[21], Cristina de O. Araújo[3], José Luis Barragan-Ramírez[22], Gergely Boros[21], Edgar Camero-Rubio[23], Melvin Cruz[24], Eva Cuesta[1,25,], Miryam Pieri Damborsky[26], Christian M. Deschodt[12], Priyadarsanan Dharma Rajan[27], Bram D'hondt[17], Alfonso Díaz Rojas[10], Kemal Dindar[19], Federico Escobar[10], Verónica R. Espinoza[1,28], José Rafael Ferrer-Paris[29,30,31], Pablo Enrique Gutiérrez Rojas[32], Zac Hemmings[9], Benjamín Hernández[33], Sarah J. Hill[9], Maurice Hoffmann[17,34], Pierre Jay-Robert[35], Kyle Lewis[11,36], Megan Lewis[37,38], Cecilia Lozano[29,39], Diego Marín-Armijos[40], Patrícia Menegaz de Farias[41], Betselene Murcia-Ordoñez[33], Seena Narayanan Karimbumkara[27], José Luis Navarrete-Heredia[22], Candelaria Ortega-Echeverría[42], José D. Pablo-Cea[43], William Perrin[35], Marcelo Bruno Pessoa[1,3], Anu Radhakrishnan[27], Iraj Rahimi[44], Amalia Teresa Raimundo[26], Diana Catalina Ramos[23], Ramón E. Rebolledo[45], Angela Roggero[18], Ada Sánchez-Mercado[29,30,46], László Somay[21], Jutta Stadler[47], Pejman Tahmasebi[44], José Darwin Triana Céspedes[33], Ana M. C. Santos[25,48,*]

* Correspondence and requests for materials should be addressed to Joaquín Hortal or Ana Margarida Coelho dos Santos. Emails: jhortal@mncn.csic.es, anamc.santos@uam.es
† These authors contributed equally to this work

Extended data file containing:
Figs. S1 to S5
Tables S1 to S7
Appendices S1, S2 and S3

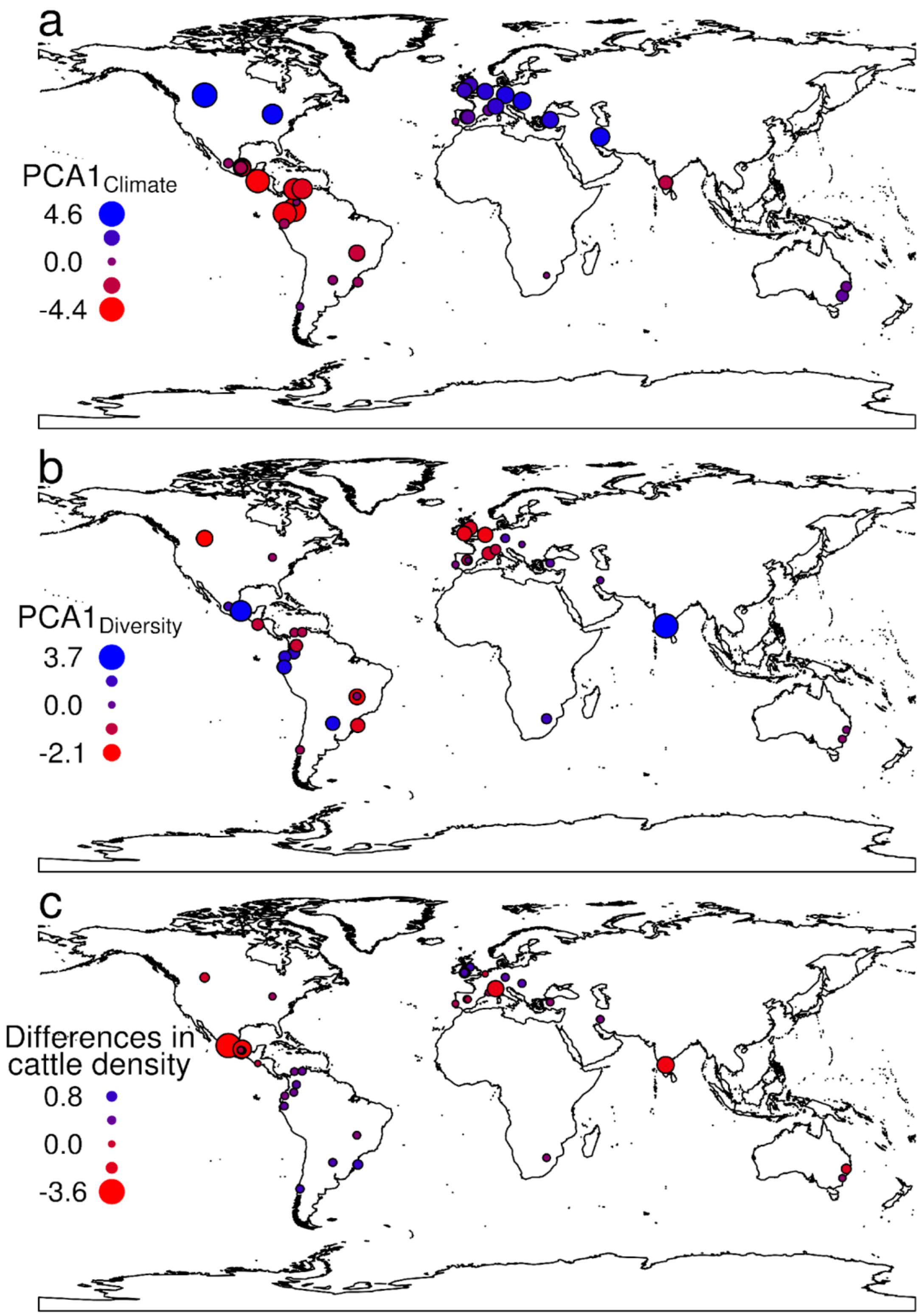

Fig. S1. Variation in climate (a), and differences in dung beetle diversity (b) and cattle density (c) between low- and high-intensity grazing treatments, standardized as z-scores. In (a), negative values indicate higher temperatures and daily temperature variations (i.e. isothermality), lower temperature seasonality and yearly temperature variations, and to a less extent, higher precipitations (see Table S5 for a full description of PCA loadings per climatic variable). In (b) and (c), negative values indicate greater diversity or cattle density in high-intensity grazing. Circle sizes indicate absolute values, and color indicates signs (blue: positive, red: negative).

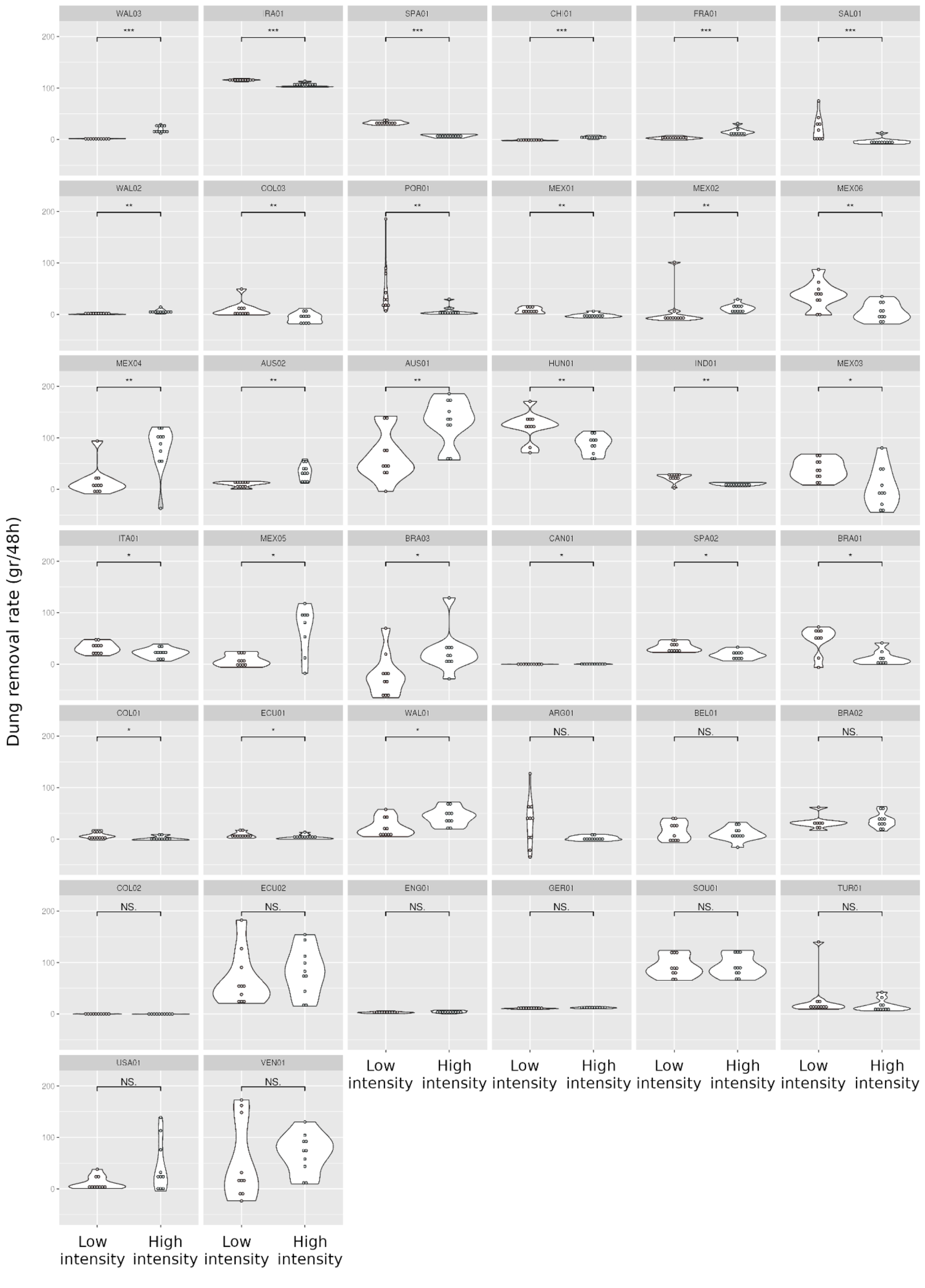

Fig. S2. **Effects of cattle management intensity (low- vs high-intensity grazing) on dung beetle dung removal rates (g/48h) in each studied landscape.** Asterisks correspond to the levels of significance of Wilcoxon signed-rank tests: * = p<0.05, ** = p<0.01, *** = p<0.001, NS=non-significant. Site codes as in Supplementary data.

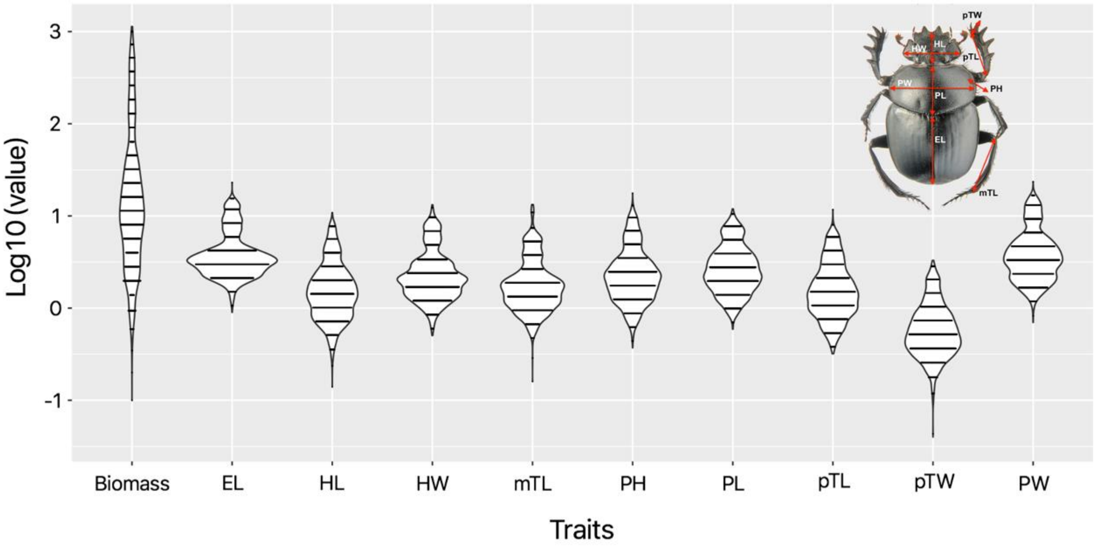

Fig. S3. Variation in the values of biomass and nine dung beetle morphological traits measured to account for Functional Diversity across all the species collected in all 38 study sites. Trait values have been Log10 transformed. Trait abbreviations stand for: Biomass=dry weight, EL=elytra length, HL=head length, HW=head width, mTL=metatibia length, PH=pronotum height, PL=pronotum length, pTL=protibia length, pTW=protibia width, and PW=pronotum width. Individual measurements can be found in the Supplementary Data.

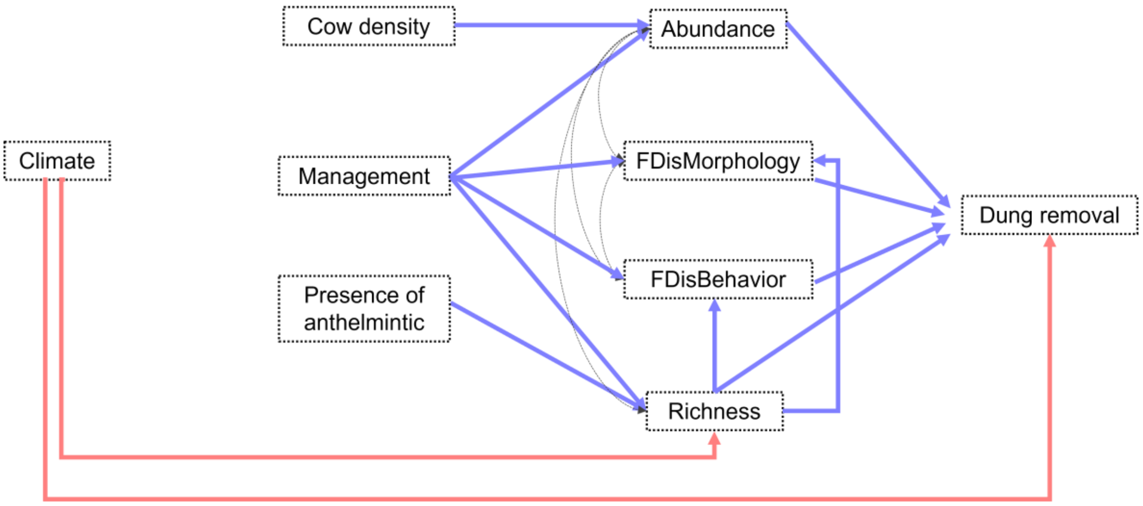

Fig S4. Prior conceptual model of the relationships between climate, different aspects of biodiversity and dung removal rates under different (low-intensity and high-intensity) cattle management regimes. Positive and negative effects are indicated by blue and red arrows, respectively; the direction of the arrows denotes the direction of the effect, curved black arrows indicate that variables have correlated errors. *FDisMorphology* and *FDisBehavior* stand for the Functional Dispersion of, respectively, morphological and behavioral dung beetle traits. See Methods for the description of variables.

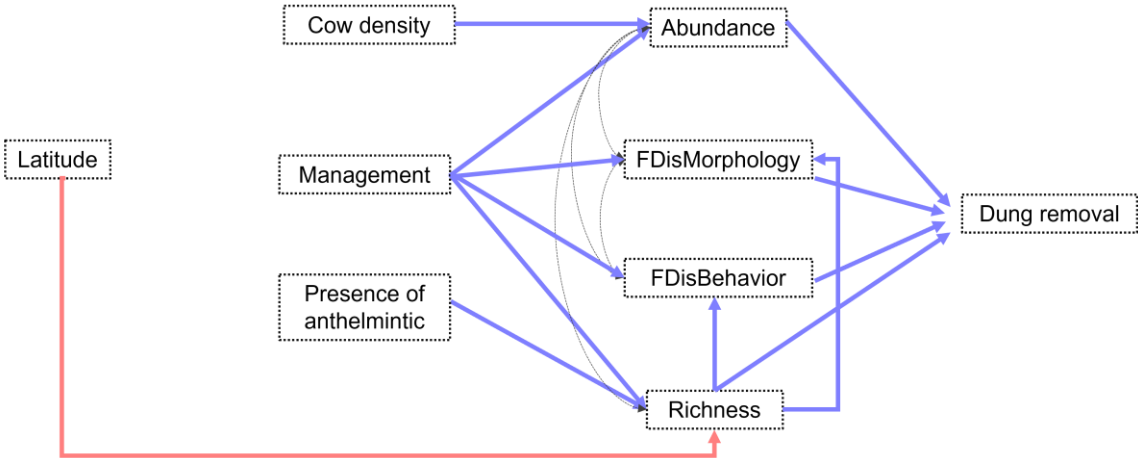

Fig S5. Prior conceptual model of the relationships between latitude, different aspects of biodiversity and dung removal rates under different (low-intensity and high-intensity) cattle management regimes. Positive and negative effects are indicated by blue and red arrows, respectively; the direction of the arrows denotes the direction of the effect, curved black arrows indicate that variables have correlated errors. *FDisMorphology* and *FDisBehavior* stand for the Functional Dispersion of, respectively, morphological and behavioral dung beetle traits. See Methods for the description of all variables.

Table S1. Results of a piecewiseSEM model to disentangle the relative importance of climate (summarized by $PCA1_{Climate}$), management regimes and anthelmintic use on diversity metrics (i.e., abundance, richness, *FDisMorphology*, and *FDisBehavior*) and dung removal rates. Biogeographical variation was considered by using mixed models and including the biogeographical region as a random factor. The final model *D*-separation one-sided test returned a Fisher's C = 20.28; $p = 0.44$; DF = 20; AIC = 254.13. Marginal R^2 stands for the variance explained by fixed factors, while Conditional R^2 stands for the variance explained both by fixed and random factors. VIF stands for variance inflation factor. Variables with ~~ symbol were included in the model with correlated errors (see Methods). The random factor was excluded from the Dung removal model since it did not explain any additional variability beyond the fixed effects (i.e., Marginal R^2 = Conditional R^2).

**Random factor**	**Response**	**Predictor**	**Estimate**	**SE**	**DF**	**Std.Estimate**	***P***	**VIF**	**Marginal R^2**	**Conditional R^2**
Biogeographical region	Abundance	Cattle Density	-0.01	0.01	70.97	-0.06	0.60		0	0.2
Biogeographical region	Abundance	Management regimes	0.03	0.39	68.72	0.01	0.95			
Biogeographical region	**Richness**	***$PCA1_{Climate}$***	-0.07	0.03	70.25	**-0.26**	**0.04**		0.05	0.68
Biogeographical region	Richness	Anthelmintic usage	-0.10	0.13	68.08	-0.07	0.46			
Biogeographical region	Richness	Management regimes	-0.16	0.11	67.05	-0.13	0.15			
Biogeographical region	***FDisBehavior***	**Richness**	0.05	0.01	47.19	**0.49**	**0.00**		0.25	0.33
Biogeographical region	*FDisBehavior*	Management regimes	-0.02	0.01	69.60	-0.14	0.16			
Biogeographical region	*FDisMorphology*	Richness	0.01	0.01	38.27	0.09	0.52	1.03	0.16	0.25
Biogeographical region	***FDisMorphology***	***$PCA1_{Climate}$***	-0.01	0.00	24.78	**-0.40**	**0.02**	1.004		
Biogeographical region	*FDisMorphology*	Management regimes	-0.01	0.01	68.67	-0.09	0.39			
None	Dung removal	Abundance	0.17	0.09	69.00	0.26	0.05	1.71	0.32	-
None	Dung removal	*FDisBehavior*	-1.92	2.40	69.00	-0.12	0.43	2.13		
None	Dung removal	*FDisMorphology*	3.01	3.72	69.00	0.11	0.42	1.92		
None	Dung removal	***$PCA1_{Climate}$***	-0.12	0.05	69.00	**-0.27**	**0.02**	1.3		
None	Dung removal	**Richness**	0.81	0.25	69.00	**0.45**	**0.00**	1.94		
None	Dung removal	Management regimes	0.17	0.23	69.00	0.07	0.46			
	~~Abundance	~~Richness	0.41	-	76.00	0.41	0.00			
	~~*FDisMorphology*	~~*FDis Behavior*	0.56	-	76.00	0.56	0.00			
	~~*FDisBehavior*.log1	~~Abundance	-0.37	-	76.00	-0.37	0.00			
	~~*FDisMorphology*	~~Abundance	-0.29	-	76.00	-0.29	0.01			

Table S2. Results of a piecewiseSEM model to disentangle the relative importance of latitude, management regimes and anthelmintic use on diversity metrics (i.e., abundance, richness, *FDisMorphology*, and *FDisBehavior*) and dung removal rates. Biogeographical variation was considered by using mixed models and including the biogeographical region as a random factor. The final model *D*-separation one-sided test returned a Fisher's C = 18.18; p = 0.58; DF = 20; AIC = 275.06. Marginal R^2 stands for the variance explained by fixed factors, while Conditional R^2 stands for the variance explained both by fixed and random factors. VIF stands for variance inflation factor. Variables with ~~ symbol were included in the model with correlated errors (see Methods). The random factor was excluded from the Dung removal model since it did not explain any additional variability beyond the fixed effects (i.e., Marginal R^2 = Conditional R^2).

Random factor	Response	Predictor	Estimate	SE	DF	Std.Estimate	*P*	VIF	Marginal R^2	Conditional R^2
Biogeographical region	Abundance	Cattle Density	-0.01	0.01	0.28	-0.06	0.60		0	0.2
Biogeographical region	Abundance	Management regimes	0.03	0.39	0.00	0.01	0.95			
Biogeographical region	Richness	Latitude	0.00	0.00	0.40	-0.13	0.53		0-03	0.63
Biogeographical region	Richness	Anthelmintic usage	-0.11	0.14	0.62	-0.07	0.44			
Biogeographical region	Richness	Management regimes	-0.16	0.11	1.95	-0.12	0.17			
Biogeographical region	***FDisBehavior***	**Richness**	0.05	0.01	15.21	**0.49**	**0.00**		0.25	0.33
Biogeographical region	*FDisBehavior*	Management regimes	-0.02	0.01	1.98	-0.14	0.16			
Biogeographical region	*FDisMorphology*	Richness	0.01	0.01	1.12	0.15	0.29	1.03	0.05	0.28
Biogeographical region	*FDisMorphology*	Latitude	0.00	0.00	1.06	-0.22	0.32	1.01		
Biogeographical region	*FDisMorphology*	Management regimes	-0.01	0.01	0.59	-0.08	0.45			
None	Dung removal	Abundance	0.12	0.09	1.32	0.18	0.19	1.75	0.28	
None	Dung removal	*FDisBehavior*	-2.03	2.44	-0.83	-0.12	0.41	2.13		
None	Dung removal	*FDisMorphology*	4.06	3.71	1.09	0.15	0.28	1.85		
None	Dung removal	Latitude	-0.01	0.00	-1.83	-0.20	0.07	1.18		
None	**Dung removal**	**Richness**	0.87	0.26	3.31	**0.48**	**0.00**	2.07		
None	Dung removal	Management regimes	0.19	0.24	0.79	0.08	0.43			
	~~Abundance	~~Richness	0.35	-	3.23	0.35	0.00			
	~~*FDisMorphology*	~~*FDisBehavior*	0.59	-	6.25	0.59	0.00			
	~~*FDisBehavior*.log1	~~Abundance	-0.37	-	-3.45	-0.37	0.00			
	~~*FDisMorphology*	~~Abundance	-0.36	-	-3.25	-0.36	0.00			

Table S3. Results of a meta-regression model assessing the effects of climate (*$PCA1_{Climate}$*) and difference in cattle density (*Δ Cattle Density*) on the difference in dung removal rates between management regimes (low-high intensities pastures). The effects of the other variables (differences in dung beetle abundance, richness, and functional diversity based on both behavioral and morphological traits) were tested in separate models. VIF stands for variance inflation factor. The significance of the meta-regression model as a whole was tested by *F*-tests, whereas the significance of each partial regression coefficient (moderator) was tested using *t*-tests. We did not applied adjustments in *p*-values because of the exploratory nature of these sensitivity analyses.

Moderators	Estimat	SE	*t*	*P*	Pseudo-	*F*	df	*P*	VIF
Intercept	0.013	0.256	0.051	0.960					
$PCA1_{Climate}$	-0.156	0.102	-1.528	0.136	0.090	1.001	3; 34	0.404	1.06
Δ Cattle Density	-0.203	0.250	-0.812	0.423					1.00
Abundance	-0.111	0.255	-0.436	0.666					1.06
Intercept	0.054	0.251	0.213	0.833					
$PCA1_{Climate}$	-0.105	0.100	-1.058	0.297	0.173	2.218	3; 34	0.104	1.06
Δ Cattle Density	-0.071	0.253	-0.280	0.781					1.14
Richness	0.455	0.251	1.817	0.078					1.20
Intercept	0.061	0.259	0.237	0.814					
$PCA1_{Climate}$	-0.119	0.101	-1.180	0.246	0.117	2.222	3; 34	0.103	1.03
Δ Cattle Density	-0.209	0.244	-0.859	0.396					1.00
FDisBehavior	0.408	0.220	1.857	0.072					1.03
Intercept	0.132	0.268	0.494	0.624					
$PCA1_{Climate}$	-0.116	0.104	-1.115	0.273	0.103	**2.994**	3; 34	0.044	1.03
Δ Cattle Density	-0.282	0.238	-1.183	0.245					1.00
FDisMorphology	0.520	0.227	**2.293**	0.028					1.03

Table S4. Results of a meta-regression model assessing the effects of latitude and difference in cattle density (*Δ Cattle Density*) on the difference in dung removal rates between management regimes (low-high intensities pastures). The effects of the other variables (differences in dung beetle abundance, richness, and functional diversity based on both behavioral and morphological traits) were tested using the scores of a principal component analysis to summarize them ($PCA1_{Diversity}$) and using each variable separately. The significance of the meta-regression model as a whole was tested by *F*-tests, whereas the significance of each partial regression coefficient (moderator) was tested using *t*-tests. We did not applied adjustments in *p*-values because of the exploratory nature of these sensitivity analyses.

Moderators	Estimate	SE	*t*	*P*	Pseudo-R^2	*F*	df	*P*	VIF
Intercept	0.816	0.536	1.521	0.137	0.192	**3.751**	3; 34	0.020	
Latitude	-0.025	0.016	-1.539	0.133					1.11
Δ Cattle Density	-0.187	0.233	-0.804	0.427					1.02
$\boldsymbol{PCA1_{Diversity}}$	0.351	0.163	**2.158**	0.038					1.12
Intercept	1.126	0.517	2.178	0.036	0.180	2.283	3; 34	0.097	
Latitude	-0.039	0.016	-2.486	0.018					1.08
Δ Cattle Density	-0.199	0.239	-0.833	0.411					1.00
Abundance	-0.162	0.248	-0.654	0.518					1.08
Intercept	0.856	0.521	1.642	0.110	0.220	2.771	3; 34	0.057	
Latitude	-0.028	0.016	-1.770	0.086					1.11
Δ Cattle Density	-0.089	0.249	-0.359	0.722					1.15
Richness	0.356	0.254	1.402	0.170					1.26
Intercept	0.911	0.524	1.737	0.092	0.182	2.862	3; 34	0.051	
Latitude	-0.029	0.016	-1.862	0.071					1.06
Δ Cattle Density	-0.205	0.236	-0.869	0.391					1.00
FDisBehavior	0.339	0.219	1.548	0.131					1.06
Intercept	0.939	0.535	1.756	0.088	0.172	**3.465**	3; 34	0.027	
Latitude	-0.028	0.016	-1.751	0.089					1.07
Δ Cattle Density	-0.269	0.232	-1.160	0.254					1.00
FDisMorphology	0.439	0.226	1.943	0.060					1.07

Table S5. Loadings, eigenvalues, and percentage of explanation for Principal Component Analysis (PCA) conducted with climatic variables. Variables with loadings greater than |0.75| are in bold.

Variables	PCA1	PCA2
Annual Mean Temperature	**-0.96**	-0.18
Mean Diurnal Range	-0.05	**-0.75**
Isothermality	**-0.88**	-0.02
Temperature Seasonality	**0.90**	-0.22
Maximum Temperature of Warmest Month	-0.57	-0.64
Minimum Temperature of Coldest Month	**-0.96**	0.11
Temperature Annual Range	**0.77**	-0.50
Mean Temperature of Wettest Quarter	**-0.77**	-0.12
Mean Temperature of Driest Quarter	**-0.77**	-0.25
Annual Precipitation	-0.67	0.53
Precipitation Seasonality	-0.62	-0.64
Precipitation of Wettest Quarter	-0.59	0.41
Precipitation of Driest Quarter	0.00	**0.85**
**Eigenvalue**	6.72	2.98
**% explanation**	51.68	22.92

Table S6. Loadings, eigenvalues, and percentage of explanation for Principal Component Analysis (PCA) conducted with dung beetle diversity indices (differences between low- and high-intensity grazing). Variables with loadings greater than |0.75| are in bold.

Diversity indices	PCA1	PCA2
Abundance	0.01	**-0.92**
Richness	0.63	-0.37
FDisBehavior	**0.92**	0.08
FDisMorphology	**0.79**	0.22
**Eigenvalue**	1.87	1.05
**% explanation**	46.78	26.13

Table S7. Assessment of different spatial correlation structures in the accumulated effect size estimate.

Spatial correlation structure	AICc
Exponential	151.41
Gaussian	152.41
Rational Quadratic	151.91
Spheric	152.14

Appendix S1. List of structural equations used to disentangle the relative importance of climate (summarized by *PCA1Climate*), management regimes and anthelmintic use on diversity metrics (i.e., abundance, richness, FDMorphology [Fdis.morph], and FDisBehavior [Fdis.behav]) and dung removal rates:

Initial model:

```
init.mod = psem(
  lmer(abun.log1 ~ cow.dens + group1 + (1|bioregion), data = df),
  lmer(rich.log1 ~ Clim_PCA1 + anthelmintic2 + group1 + (1|bioregion), data = df),
  lmer(Fdis.behav.log1 ~ rich.log1 + group1 + (1|bioregion), data = df),
  lmer(Fdis.morph.log1 ~ rich.log1 + group1 + (1|bioregion), data = df),
  lmer(mean.removal.log1 ~ abun.log1 + Fdis.behav.log1 + Fdis.morph.log1 + Clim_PCA1 +
group1 + (1|bioregion), data = df),
  abun.log1 %~~% rich.log1,
  Fdis.morph.log1 %~~% Fdis.behav.log1,
  Fdis.behav.log1 %~~% abun.log1,
  Fdis.morph.log1 %~~% abun.log1)
```

Final model:

```
fin.mod = psem(
  lmer(abun.log1 ~ cow.dens + group1 + (1|bioregion), data = df),
  lmer(rich.log1 ~ Clim_PCA1 + anthelmintic2 + group1 + (1|bioregion), data = df),
  lmer(Fdis.behav.log1 ~ rich.log1 + group1 + (1|bioregion), data = df),
  lmer(Fdis.morph.log1 ~ rich.log1 + group1 + Clim_PCA1 + (1|bioregion), data = df),
  lm(mean.removal.log1 ~ abun.log1 + Fdis.behav.log1 + Fdis.morph.log1 + Clim_PCA1 +
rich.log1 + group1, data = df),
  abun.log1 %~~% rich.log1,
  Fdis.morph.log1 %~~% Fdis.behav.log1,
  Fdis.behav.log1 %~~% abun.log1,
  Fdis.morph.log1 %~~% abun.log1)
```

Appendix S2. List of structural equations used to disentangle the relative importance of latitude, management regimes and anthelmintic use on diversity metrics (i.e., abundance, richness, FDMorphology [Fdis.morph], and FDisBehavior [Fdis.behav]) and dung removal rates:

Initial model:

```
init.mod = psem(
  lmer(abun.log1 ~ cow.dens + group1 + (1|bioregion), data = df),
  lmer(rich.log1 ~ lat + anthelmintic2 + group1 + (1|bioregion), data = df),
  lmer(Fdis.behav.log1 ~ rich.log1 + group1 + (1|bioregion), data = df),
  lmer(Fdis.morph.log1 ~ rich.log1 + group1 + (1|bioregion), data = df),
  lmer(mean.removal.log1 ~ abun.log1 + Fdis.behav.log1 + Fdis.morph.log1 + group1 +
(1|bioregion), data = df),
  abun.log1 %~~% rich.log1,
  Fdis.morph.log1 %~~% Fdis.behav.log1,
  Fdis.behav.log1 %~~% abun.log1,
  Fdis.morph.log1 %~~% abun.log1)
```

Final model:

```
fin.mod = psem(
  lmer(abun.log1 ~ cow.dens + group1 + (1|bioregion), data = df),
  lmer(rich.log1 ~ lat + anthelmintic2 + group1 + (1|bioregion), data = df),
  lmer(Fdis.behav.log1 ~ rich.log1 + group1 + (1|bioregion), data = df),
  lmer(Fdis.morph.log1 ~ rich.log1 + group1 + (1|bioregion), data = df),
  lmer(mean.removal.log1 ~ abun.log1 + Fdis.behav.log1 + Fdis.morph.log1 + rich.log1 + group1
+ (1|bioregion), data = df),
  abun.log1 %~~% rich.log1,
  Fdis.morph.log1 %~~% Fdis.behav.log1,
  Fdis.behav.log1 %~~% abun.log1,
  Fdis.morph.log1 %~~% abun.log1)
```

Appendix S3. Script for meta-analytical models

##Important for interpretation: in our datasets and script, LOW-intensity #

#treatment is coded as "EXTENSIVE" and HIGH-intensity treatment is coded #as "INTENSIVE". #

##dung_data<-read.csv("dung_data.csv",h=T,sep=",")#Import dung-beetle data

#Import packages:

library(metafor)

library(extrafont)

#Compute Hedges' g:

es_dung<-escalc(measure = "SMD", m1i = mean.removal.extensive, sd1i = sd.removal.extensive, n1i = n.removal.extensive, m2i = mean.removal.intensive, sd2i = sd.removal.intensive, n2i = n.removal.intensive, data = dung_data,append=F)

rownames(es_dung)<-dung_data$site #Name rows with site codes

#Positive g values means that there is a higher manure removal in #extensive (low-intensity treatment) than intensive (high-intensity #treatment) treatments. We can see the positive sign in the numerator of #Hedges' g:

dung_data$mean.removal.extensive[1] - dung_data$mean.removal.intensive[1]

#Descriptive statistics and data exploration:

summary(es_dung$yi) #Summary of effect-size

sd(es_dung$yi) #SD of effect-size

sum(summary(es_dung)$ci.lb > 0) #Number of positive effect-sizes

sum(summary(es_dung)$ci.ub < 0) #Number of negative effect-sizes

sum(summary(es_dung)$ci.lb < 0 & summary(es_dung)$ci.ub > 0)#Number #of non-significant effect-sizes

#############################

####Preparation of moderators datasets###

#############################

#We separate the data in different datasets to ease manipulation:

geogr<-dung_data[,41:43] #Biogeographic region and site coordinates

climate<-dung_data[,22:40] #Climate data

#We filtered climate variables only for those variables relevant for dung #beetles:

```
climate <- climate[,c(1:9,12,15,17,18)] #"BIO.1", "BIO.2", "BIO.3", "BIO.4",

#"BIO.5", "BIO.6", "BIO.7", "BIO.8", "BIO.9", "BIO.12","BIO.15", "BIO.17" and #"BIO.18"

colnames(climate) <- c("AMT", "MDR", "ISO", "TS", "MTWM", "MTCM", "TAR", "MTWQ", "MTDQ", "AP", "PS", "PDQ", "PWQ") #We renamed the bioclimatic #variables with their respective codes

head(climate) #Just checking

#We computed differences in cattle density:

diff_cow.dens <- dung_data$cow.dens.extensive - dung_data$cow.dens.intensive

#Similar to Hedges' g, positive values of differences in cattle density means #that there is a higher cattle density in extensive (low-intensity treatment) #than intensive (high-intensity treatment) treatments

#We summarized climate variables with a PCA:

require(vegan)

pca_clim<-prcomp(climate, scale. = T)

eigen_vals_clim<-apply(pca_clim$x,2,var) #Eigenvalues from Climate PCA

round(eigen_vals_clim,2)

pca_load_clim<-cor(climate,pca_clim$x[,1:2]) #Loadings from Climate PCA

round(pca_load_clim,2)

round(100*(eigen_vals_clim/sum(eigen_vals_clim)),2) #Percent of #explanation of each axis

#We filtered only those loadings with values > |.75|:

pca_load_clim_sig <- pca_load_clim[(abs(pca_load_clim[,1])>.75 | abs(pca_load_clim[,2])>.75),]

pca_load_clim_sig <- round(pca_load_clim_sig,2)

pca_load_clim_sig

#We filtered dung beetle diversity indexes:

div_ext<-dung_data[,c(8,9,11,13)] #Diversity data from Extensive (low-#intensity) treatment

div_int<-dung_data[,c(15,16,18,20)]  #Diversity data from Intensive (high-#intensity) treatment

colnames(div_ext)<-c("abun","rich","Fdis.behav","Fdis.morph")

colnames(div_int)<-c("abun","rich","Fdis.behav","Fdis.morph")

#Differences in dung-beetle diversity for each experimental site:

diff_div<-div_ext-div_int #Computed the difference in

#diversity indexes between extensive (low) and intensive (high-intensity) #treatments

#PCA of differences in diversity:
```

```
pca_div <- prcomp(diff_div, scale. = T)

pca_div_autovals <- apply(pca_div$x,2,var) #Eigenvalues

pca_div_autovals

pca_div_pexp <- (pca_div_autovals/sum(pca_div_autovals))*100 #Proportion #of explanation

pca_div_pexp

pca_load_div<-cor(diff_div,pca_div$x[,1:2]) #Loadings

round(pca_load_div,2)

##############################

#Accumulated effect size and meta-regression#

##############################

#We prepared the matrix with moderator variables and experiments #coordinates:

preds<-data.frame(Clim_PCA1 = pca_clim$x[,1], #Clim_PCA1 contains the #scores from PCA1 conducted with climate variables

                diff_cow.dens = decostand(diff_cow.dens, "stan"), #diff_cow.dens contains the standardized differences in cattle density

                diff_Div_PCA1 = pca_div$x[,1], #diff_Div_PCA1 contains the #scores from PCA1 conducted with differences in diversity indexes

                lon = geogr$lon, lat = geogr$lat) #lon and lat are the #coordinates of each experiment

#Differences in cattle density was standardized to approximate to the scale #of PCA scores

cor_mods<-as.dist(round(cor(preds[,1:3]),2))

cor_mods#All correlations among moderators are < |.35|

#Accumulated effect size with spatial structure:

group<-rep("a",nrow(es_dung))

accu_exp<-rma.mv(es_dung$yi, V = es_dung$vi, data = preds, method = "ML", test = "t", struct = "SPEXP", random = ~ lon + lat|group)


accu_gau<-rma.mv(es_dung$yi, V = es_dung$vi, data = preds, method = "ML", test = "t", struct = "SPGAU", random = ~ lon + lat|group)

accu_rat<-rma.mv(es_dung$yi, V = es_dung$vi, data = preds, method = "ML", test = "t", struct = "SPRAT", random = ~ lon + lat|group)

accu_sph<-rma.mv(es_dung$yi, V = es_dung$vi, data = preds, method = "ML", test = "t", struct = "SPSPH", random = ~ lon + lat|group)

fitstats(accu_exp)[5];fitstats(accu_gau)[5];
```

```
fitstats(accu_rat)[5];fitstats(accu_sph)[5]

accu_exp#Presented the lowest AICc

#I2 for rma.mv following Nakagawa & Santos 2012:

s2_fun<-function(vi,data){
  wi<-1/vi
  wi.2<-wi^2
  k<-nrow(data)
  fst.ter<-sum((k-1)*wi,na.rm = T)
  snd.ter<-(sum(wi,na.rm = T)^2)-(sum(wi.2,na.rm = T))
  s2_obs<-fst.ter/snd.ter
  return(s2_obs)
}

error<-s2_fun(vi=es_dung$vi,data=es_dung)

#I2 (Nakagawa & Santos 2012):

round(sum(accu_exp$tau2)/(sum(accu_exp$tau2)+error),3)

#Meta-regression

#Exponential correlation structure:

rma_exp<-rma.mv(es_dung$yi ~ Clim_PCA1 + diff_cow.dens + diff_Div_PCA1, V = es_dung$vi,
data = preds, method = "ML", test = "t", struct = "SPEXP", random = ~ lon + lat|group)

#Gaussian correlation structure:

rma_gau<-rma.mv(es_dung$yi ~ Clim_PCA1 + diff_cow.dens + diff_Div_PCA1, V = es_dung$vi,
data = preds, method = "ML", test = "t", struct = "SPGAU", random = ~ lon + lat|group)

#Rational Quadratic correlation structure:

rma_rat<-rma.mv(es_dung$yi ~ Clim_PCA1 + diff_cow.dens + diff_Div_PCA1, V = es_dung$vi,
data = preds, method = "ML", test = "t", struct = "SPRAT", random = ~ lon + lat|group)

#Spherical correlation structure:

rma_sph<-rma.mv(es_dung$yi ~ Clim_PCA1 + diff_cow.dens + diff_Div_PCA1, V = es_dung$vi,
data = preds, method = "ML", test = "t", struct = "SPSPH", random = ~ lon + lat|group)

fitstats(rma_exp)[5];fitstats(rma_gau)[5];

fitstats(rma_rat)[5];fitstats(rma_sph)[5]

rma_exp #Exponential structure presented the lowest AIC

#Pseudo-R2 following Borenstein et al. 2009:
```

```
1-(rma_exp$tau2/accu_exp$tau2)

#VIF:

vif(rma_exp)

######

#Figures#

######

#Map of effect-size variation:

require(RColorBrewer); require(plotrix);require(maptools)

require(rgeos)

data("wrld_simpl")

wbuf = gBuffer(wrld_simpl,width=0.00001)

#We created an object that represent the magnitude of effect in an #absolute scale:

size_es<-abs(as.numeric(es_dung$yi))

size_es<-(((size_es-min(size_es))/(max(size_es)-min(size_es)))*1.5)+.5 #Symbols size ranging from .5 to 2

#We colored each effect size to represent the sign of Hedges' g difference #with red meaning higher manure removal in intensive (high-intensity) #treatment, and blue higher removal in extensive (low-intensity) treatment:

col.fun_ES<-colorRampPalette(c("red","white","blue"))(length(es_dung$yi))

col.fun_ES<-col.fun_ES[with(es_dung, findInterval(yi, sort(unique(yi))))]

#Join the effect size, its symbol size and color in a single data-frame to ease

#manipulation below:

ES_col<-data.frame(es=es_dung$yi,size=size_es,color=col.fun_ES)

summary(ES_col[,1])

ES_col[ES_col$es==max(ES_col$es),] #The highest value

ES_col[29,]

ES_col[4,] #Closet to the mean effect

ES_col[8,]

ES_col[ES_col$es==min(ES_col$es),]#The lowest value

#Figure 3a:

tiff("Fig3a_Map_EffectSize.tiff",res=600,compression="lzw",width = 3500,height = 3500)

par(mar=c(0,0,0,0)+.1)
```

```
plot(wbuf,col="grey90")

points(preds$lon, preds$lat, cex=ES_col$size, pch=21,
bg=rgb(t(col2rgb(ES_col$color))/255,alpha = .5))

polygon(x=c(-192,-114,-114,-192),y=c(23,23,-55,-55),col="white",border = F)

points(x=rep(-150,5), y=c(0,-10,-20,-30,-40), cex=ES_col$size[c(31,29,4,8,38)],
bg=rgb(t(col2rgb(ES_col$color))/255, alpha = .5)[c(31,29,4,8,38)], pch=21)

text(x=rep(-170,3),y=c(0,-20,-40),c("3.4","-0.1","-4.7"),cex=1.05)

text(x=-155,y=15,"Hedges' g",cex=1.15)

text(x=-175,y=80,"a",cex=1.75)

dev.off()

#Fig 3 two lower panels:

#Join data for figure:

es_dung_ord <- data.frame(es_dung,wi=weights(rma_exp), size=(weights(rma_exp)-
min(weights(rma_exp)))/(max(weights(rma_exp))-min(weights(rma_exp))))

es_dung_ord <- es_dung_ord[order(es_dung_ord$yi,decreasing = T),]

#es_dung_ord is ordered by increasing effect size values

tiff("Fig3b_forest_plot.tiff",res=600,compression="lzw",width = 3500,height = 3000)

par(las=1,mar=c(5,5,.5,.5)+.1)

plot(es_dung_ord$yi,y = seq(from=1,to=length(es_dung_ord$yi)),xlim=c(-7,6),
cex.axis=1.5,xlab="Hedges' g",ylab="Sites ranked by effect sizes",
pch=22,bg="black",cex=es_dung_ord$size*3+.5,axes=F,cex.lab=1.75, ylim=c(-1,40))

arrows(x0 = es_dung_ord$yi,y0 = seq(from=1,to=length(es_dung_ord$yi)),
x1=c(es_dung_ord$yi-(1.96*sqrt(es_dung_ord$vi)), es_dung_ord$yi +
(1.96*sqrt(es_dung_ord$vi))), length = 0)

axis(side=1,at=c(-10,-6,-4,-2,0,2,4,6),cex.axis=1.5)

axis(side=2,at=c(-100,0,10,20,30,40),cex.axis=1.5)

abline(v=0,lty=2)

polygon(x=c(accu_exp$ci.lb,as.numeric(accu_exp$b),accu_exp$ci.ub,
as.numeric(accu_exp$b)), y=c(-1,-2,-1,0),col="black")

text("b",x=-7,y=38,cex=1.75)

dev.off()

size_rma <-  (weights(rma_exp) – min(weights(rma_exp))) / (max(weights(rma_exp)) -
min(weights(rma_exp)))

size_rma <- (size_rma*3)+1.5
```

```
tiff("Fig3c_meta_regression.tiff",res=600,compression="lzw",width = 3500,height = 3000)

par(las=1,mar=c(5,5,.5,.5)+.1)

regplot(rma_exp,mod = "diff_Div_PCA1", xlab=expression(PCA1[Diversity]), ylab="Hedges' g", ci=F, bg="white", xlim=c(-2,4), ylim=c(-5,4), cex.axis=1.5, cex.lab=1.75, psize=size_rma)

text("c",x=-2,y=4,cex=1.75)

dev.off()

#Supplementary figures:

#Map PCA1 Climate:

size_clim<-abs(as.numeric(preds$Clim_PCA1))

size_clim<-(((size_clim-min(size_clim))/(max(size_clim)-min(size_clim)))*1.5)+.5

col.fun_clim<-colorRampPalette(c("red","blue"))(length(preds$Clim_PCA1))

col.fun_clim<-col.fun_clim[with(preds, findInterval(Clim_PCA1, sort(unique(Clim_PCA1))))]

clim_col <- data.frame(clim=preds$Clim_PCA1, size=size_clim, color=col.fun_clim)

round(summary(clim_col[,1]),1)

clim_col

summary(clim_col$clim)

clim_col[order(clim_col$clim),]

clim_col[clim_col$clim==max(clim_col$clim),]

clim_col[15,]

clim_col[11,] #Closet to the mean effect

clim_col[5,]

clim_col[clim_col$clim==min(clim_col$clim),]

tiff("FigS1a_map_clim.tiff",res=600,compression="lzw",width = 3500,height = 3500)

par(mar=c(0,0,0,0)+.1)

plot(wbuf)

points(preds$lon, preds$lat, cex=clim_col$size, pch=21, bg=as.character(clim_col$color))

polygon(x=c(-192,-114,-114,-192), y=c(23,23,-55,-55), col="white", border = F)

points(x=rep(-150,5), y=c(0,-10,-20,-30,-40), cex=clim_col$size[c(8,15,11,5,10)], col=as.character(clim_col$color[c(8,15,11,5,10)]), pch=19)

text(x=rep(-170,3), y=c(0,-20,-40), c("4.6","0.0","-4.4"), cex=1.05)

text(x=-155, y=15, expression("PCA1"[Climate]), cex=1.15)

text("a", x=-175, y=80, cex=1.75)
```

```
dev.off()

#Map PCA1 Diversity:

size_div <- abs(as.numeric(preds$diff_Div_PCA1))

size_div <- (((size_div – min(size_div)) / (max(size_div) – min(size_div))) * 1.5) + .5

col.fun_div<-colorRampPalette(c("red","blue"))(length(preds$diff_Div_PCA1))

col.fun_div<-col.fun_div[with(preds, findInterval(diff_Div_PCA1, sort(unique(diff_Div_PCA1))))]

div_col <- data.frame(div=preds$diff_Div_PCA1, size=size_div, color=col.fun_div)

round(summary(div_col[,1]),1)

div_col

summary(div_col$div)

div_col[order(div_col$div),]

div_col[div_col$div==max(div_col$div),]

div_col[30,]

div_col[18,] #Closet to the mean effect

div_col[21,]

div_col[div_col$div==min(div_col$div),]

tiff("FigS1b_map_div.tiff",res=600,compression="lzw",width = 3500,height = 3500)

par(mar=c(0,0,0,0)+.1)

plot(wbuf)

points(preds$lon, preds$lat, cex=div_col$size, pch=21, bg=as.character(div_col$color))

polygon(x=c(-192,-114,-114,-192), y=c(23,23,-55,-55), col="white", border = F)

points(x=rep(-150,5), y=c(0,-10,-20,-30,-40), cex=div_col$size[c(19,30,18,21,8)],
col=as.character(div_col$color[c(19,30,18,21,8)]), pch=19)

text(x=rep(-170,3), y=c(0,-20,-40), c("3.7","0.0","-2.1"), cex=1.05)

text(x=-155, y=15, expression("PCA1"[Diversity]), cex=1.15)

text("b",x=-175,y=80,cex=1.75)

dev.off()

#Map cattle density:

size_dens<-abs(as.numeric(preds$diff_cow.dens))

size_dens <- (((size_dens – min(size_dens)) / (max(size_dens) – min(size_dens))) * 1.5) + .5

col.fun_dens <- colorRampPalette(c("red","blue"))(length(preds$diff_cow.dens))
```

```
col.fun_dens <- col.fun_dens[with(preds, findInterval(diff_cow.dens, sort(unique(diff_cow.dens))))]

dens_col <- data.frame(dens=preds$diff_cow.dens, size=size_dens, color=col.fun_dens)

round(summary(dens_col[,1]),1)

dens_col

summary(dens_col$dens)

dens_col[order(dens_col$dens),]

dens_col[dens_col$dens==max(dens_col$dens),]

dens_col[35,]

dens_col[4,] #Closet to the mean effect

dens_col[36,]

dens_col[dens_col$dens==min(dens_col$dens),]

tiff("FigS1c_map_dens.tiff",res=600,compression="lzw",width = 3500,height = 3500)

par(mar=c(0,0,0,0)+.1)

plot(wbuf)

points(preds$lon, preds$lat, cex=dens_col$size, pch=21, bg=as.character(dens_col$color))

polygon(x=c(-192,-114,-114,-192), y=c(23,23,-55,-55), col="white", border = F)

points(x=rep(-150,5), y=c(0,-10,-20,-30,-40), cex=dens_col$size[c(7,35,4,36,22)], col=as.character(dens_col$color[c(7,35,4,36,22)]), pch=19)

text(x=rep(-170,3),y=c(0,-20,-40),c("0.8","0.0","-3.6"),cex=1.05)

text(x=-155,y=25,"Differences in",cex=1.15)

text(x=-155,y=12.5,"cattle density",cex=1.15)

text("c",x=-175,y=80,cex=1.75)

dev.off()

##############################

#Review 1 Nature Communications reanalyses#

##############################

#Analyses following reviewer comments exloring the effect of each diversity #index:

preds_Review1<-data.frame(Clim_PCA1 = pca_clim$x[,1],

                    diff_cow.dens = decostand(diff_cow.dens, "stan"),

                    diff_Div_PCA1 = pca_div$x[,1],

                    abun = decostand(diff_div$abun,"stan"),
```

```
                    rich = decostand(diff_div$rich,"stan"),
                    Fdis.behav = decostand(diff_div$Fdis.behav,"stan"),
                    Fdis.morph = decostand(diff_div$Fdis.morph,"stan"),
                    lon = geogr$lon, lat = geogr$lat)
cor_mods_Review1<-as.dist(round(cor(preds_Review1),2))
cor_mods_Review1#All correlations are weak to moderate besides Fdis #indexes
##########################################
#Considering reviewer comment to add each diversity index instead#
#of PCA1 with all four diversity indexes (see parameter estimates#
#in Table S3)                                                    #
##########################################
#Abundance
#Exponential correlation structure:
rma_exp_Review1_abun<-rma.mv(es_dung$yi ~ Clim_PCA1 + diff_cow.dens + abun, V = es_dung$vi, data = preds_Review1, method = "ML", test = "t", struct = "SPEXP", random = ~ lon + lat|group)
#Gaussian correlation structure:
rma_gau_Review1_abun<-rma.mv(es_dung$yi ~ Clim_PCA1 + diff_cow.dens + abun, V = es_dung$vi, data = preds_Review1, method = "ML", test = "t", struct = "SPGAU", random = ~ lon + lat|group)
#Rational Quadratic correlation structure:
rma_rat_Review1_abun<-rma.mv(es_dung$yi ~ Clim_PCA1 + diff_cow.dens + abun, V = es_dung$vi, data = preds_Review1, method = "ML", test = "t", struct = "SPRAT", random = ~ lon + lat|group)
#Spherical correlation structure:
rma_sph_Review1_abun<-rma.mv(es_dung$yi ~ Clim_PCA1 + diff_cow.dens + abun, V = es_dung$vi, data = preds_Review1, method = "ML", test = "t", struct = "SPSPH", random = ~ lon + lat|group)
#VIF:
vif(rma_exp_Review1_abun);vif(rma_gau_Review1_abun);
vif(rma_rat_Review1_abun);vif(rma_sph_Review1_abun)
fitstats(rma_exp_Review1_abun)[5];fitstats(rma_gau_Review1_abun)[5];
fitstats(rma_rat_Review1_abun)[5];fitstats(rma_sph_Review1_abun)[5]
rma_exp_Review1_abun #Exponential structure presented the lowest AIC
```

#Pseudo-R2 following Borenstein et al. 2009:

1-(rma_exp_Review1_abun$tau2/accu_exp$tau2)

#Richness

#Exponential correlation structure:

rma_exp_Review1_rich<-rma.mv(es_dung$yi ~ Clim_PCA1 + diff_cow.dens + rich, V = es_dung$vi, data = preds_Review1, method = "ML", test = "t", struct = "SPEXP", random = ~ lon + lat|group)

#Gaussian correlation structure:

rma_gau_Review1_rich<-rma.mv(es_dung$yi ~ Clim_PCA1 + diff_cow.dens + rich, V = es_dung$vi, data = preds_Review1, method = "ML", test = "t", struct = "SPGAU", random = ~ lon + lat|group)

#Rational Quadratic correlation structure:

rma_rat_Review1_rich<-rma.mv(es_dung$yi ~ Clim_PCA1 + diff_cow.dens + rich, V = es_dung$vi, data = preds_Review1, method = "ML", test = "t", struct = "SPRAT", random = ~ lon + lat|group)

#Spherical correlation structure:

rma_sph_Review1_rich<-rma.mv(es_dung$yi ~ Clim_PCA1 + diff_cow.dens + rich, V = es_dung$vi, data = preds_Review1, method = "ML", test = "t", struct = "SPSPH", random = ~ lon + lat|group)

#VIF:

vif(rma_exp_Review1_rich);vif(rma_gau_Review1_rich);

vif(rma_rat_Review1_rich);vif(rma_sph_Review1_rich)

fitstats(rma_exp_Review1_rich)[5];fitstats(rma_gau_Review1_rich)[5];

fitstats(rma_rat_Review1_rich)[5];fitstats(rma_sph_Review1_rich)[5]

rma_exp_Review1_rich #Exponential structure presented the lowest AIC

#Pseudo-R2 following Borenstein et al. 2009:

1-(rma_exp_Review1_rich$tau2/accu_exp$tau2)

#Fdis.behav

#Exponential correlation structure:

rma_exp_Review1_Fdis.behav<-rma.mv(es_dung$yi ~ Clim_PCA1 + diff_cow.dens + Fdis.behav, V = es_dung$vi, data = preds_Review1, method = "ML", test = "t", struct = "SPEXP", random = ~ lon + lat|group)

#Gaussian correlation structure:

rma_gau_Review1_Fdis.behav<-rma.mv(es_dung$yi ~ Clim_PCA1 + diff_cow.dens + Fdis.behav, V = es_dung$vi, data = preds_Review1, method = "ML", test = "t", struct = "SPGAU", random = ~ lon + lat|group)

#Rational Quadratic correlation structure:

rma_rat_Review1_Fdis.behav<-rma.mv(es_dung$yi ~ Clim_PCA1 + diff_cow.dens + Fdis.behav, V = es_dung$vi, data = preds_Review1, method = "ML", test = "t", struct = "SPRAT", random = ~ lon + lat|group)

#Spherical correlation structure:

rma_sph_Review1_Fdis.behav<-rma.mv(es_dung$yi ~ Clim_PCA1 + diff_cow.dens + Fdis.behav, V = es_dung$vi, data = preds_Review1, method = "ML", test = "t", struct = "SPSPH", random = ~ lon + lat|group)

#VIF:

vif(rma_exp_Review1_Fdis.behav);vif(rma_gau_Review1_Fdis.behav);

vif(rma_rat_Review1_Fdis.behav);vif(rma_sph_Review1_Fdis.behav)

fitstats(rma_exp_Review1_Fdis.behav)[5];fitstats(rma_gau_Review1_Fdis.behav)[5];

fitstats(rma_rat_Review1_Fdis.behav)[5];fitstats(rma_sph_Review1_Fdis.behav)[5]

rma_exp_Review1_Fdis.behav #Exponential structure presented the lowest #AIC

#Pseudo-R2 following Borenstein et al. 2009:

1-(rma_exp_Review1_Fdis.behav$tau2/accu_exp$tau2)

#Fdis.morph

#Exponential correlation structure:

rma_exp_Review1_Fdis.morph<-rma.mv(es_dung$yi ~ Clim_PCA1 + diff_cow.dens + Fdis.morph, V = es_dung$vi, data = preds_Review1, method = "ML", test = "t", struct = "SPEXP", random = ~ lon + lat|group)

#Gaussian correlation structure:

rma_gau_Review1_Fdis.morph<-rma.mv(es_dung$yi ~ Clim_PCA1 + diff_cow.dens + Fdis.morph, V = es_dung$vi, data = preds_Review1, method = "ML", test = "t", struct = "SPGAU", random = ~ lon + lat|group)

#Rational Quadratic correlation structure:

rma_rat_Review1_Fdis.morph<-rma.mv(es_dung$yi ~ Clim_PCA1 + diff_cow.dens + Fdis.morph, V = es_dung$vi, data = preds_Review1, method = "ML", test = "t", struct = "SPRAT", random = ~ lon + lat|group)

#Spherical correlation structure:

rma_sph_Review1_Fdis.morph<-rma.mv(es_dung$yi ~ Clim_PCA1 + diff_cow.dens + Fdis.morph, V = es_dung$vi, data = preds_Review1, method = "ML", test = "t", struct = "SPSPH", random = ~ lon + lat|group)

```
#VIF:

vif(rma_exp_Review1_Fdis.morph);vif(rma_gau_Review1_Fdis.morph);

vif(rma_rat_Review1_Fdis.morph);vif(rma_sph_Review1_Fdis.morph)

fitstats(rma_exp_Review1_Fdis.morph)[5];fitstats(rma_gau_Review1_Fdis.morph)[5];

fitstats(rma_rat_Review1_Fdis.morph)[5];fitstats(rma_sph_Review1_Fdis.morph)[5]

rma_exp_Review1_Fdis.morph #Exponential structure presented the lowest #AIC

#Pseudo-R2 following Borenstein et al. 2009:

1-(rma_exp_Review1_Fdis.morph$tau2/accu_exp$tau2)

#Analyses done in the first version:

cor_mods_Review1<-as.dist(round(cor(preds_Review1),2))

cor_mods_Review1#All correlations among moderators are < |.35| (ignore lon and lat)

#Meta-regression

#Exponential correlation structure:

rma_exp_Review1<-rma.mv(es_dung$yi ~ Clim_PCA1 + diff_cow.dens + diff_Div_PCA1, V = es_dung$vi, data = preds_Review1, method = "ML", test = "t", struct = "SPEXP", random = ~ lon + lat|group)

#Gaussian correlation structure:

rma_gau_Review1<-rma.mv(es_dung$yi ~ Clim_PCA1 + diff_cow.dens + diff_Div_PCA1, V = es_dung$vi, data = preds_Review1, method = "ML", test = "t", struct = "SPGAU", random = ~ lon + lat|group)

#Rational Quadratic correlation structure:

rma_rat_Review1<-rma.mv(es_dung$yi ~ Clim_PCA1 + diff_cow.dens + diff_Div_PCA1, V = es_dung$vi, data = preds_Review1, method = "ML", test = "t", struct = "SPRAT", random = ~ lon + lat|group)

#Spherical correlation structure:

rma_sph_Review1<-rma.mv(es_dung$yi ~ Clim_PCA1 + diff_cow.dens + diff_Div_PCA1, V = es_dung$vi, data = preds_Review1, method = "ML", test = "t", struct = "SPSPH", random = ~ lon + lat|group)

#VIF:

vif(rma_exp_Review1);vif(rma_gau_Review1);

vif(rma_rat_Review1);vif(rma_sph_Review1)

fitstats(rma_exp_Review1)[5];fitstats(rma_gau_Review1)[5];

fitstats(rma_rat_Review1)[5];fitstats(rma_sph_Review1)[5]

rma_exp_Review1 #Exponential structure presented the lowest AIC
```

```
#Pseudo-R2 following Borenstein et al. 2009:

1-(rma_exp_Review1$tau2/accu_exp$tau2)

#############################################

#Considering Reviewer comment about adding latitude instead of scores#

#from PCA1 conducted with climate variables (parameters estimates in  #

#Table S4)                                                                          #

############################################

#Meta-regression

#Exponential correlation structure:

rma_exp_Review1_lat<-rma.mv(es_dung$yi ~ abs(lat) + diff_cow.dens + diff_Div_PCA1, V = es_dung$vi, data = preds_Review1, method = "ML", test = "t", struct = "SPEXP", random = ~ lon + lat|group)

#Gaussian correlation structure:

rma_gau_Review1_lat<-rma.mv(es_dung$yi ~ abs(lat) + diff_cow.dens +  diff_Div_PCA1, V = es_dung$vi, data = preds_Review1, method = "ML", test = "t", struct = "SPGAU", random = ~ lon + lat|group)

#Rational Quadratic correlation structure:

rma_rat_Review1_lat<-rma.mv(es_dung$yi ~ abs(lat) + diff_cow.dens +  diff_Div_PCA1, V = es_dung$vi, data = preds_Review1, method = "ML", test = "t", struct = "SPRAT", random = ~ lon + lat|group)

#Spherical correlation structure:

rma_sph_Review1_lat<-rma.mv(es_dung$yi ~ abs(lat) + diff_cow.dens + diff_Div_PCA1, V = es_dung$vi, data = preds_Review1, method = "ML", test = "t", struct = "SPSPH", random = ~ lon + lat|group)

#VIF:

vif(rma_exp_Review1_lat);vif(rma_gau_Review1_lat);

vif(rma_rat_Review1_lat);vif(rma_sph_Review1_lat)

fitstats(rma_exp_Review1_lat)[5];fitstats(rma_gau_Review1_lat)[5];

fitstats(rma_rat_Review1_lat)[5];fitstats(rma_sph_Review1_lat)[5]

rma_exp_Review1_lat #Exponential structure presented the lowest AIC

#Pseudo-R2 following Borenstein et al. 2009:

1-(rma_exp_Review1_lat$tau2/accu_exp$tau2)

#Meta-regression with each diversity index

#Abundance
```

#Exponential correlation structure:

rma_exp_Review1_abun_lat<-rma.mv(es_dung$yi ~ abs(lat) + diff_cow.dens + abun, V = es_dung$vi, data = preds_Review1, method = "ML", test = "t", struct = "SPEXP", random = ~ lon + lat|group)

#Gaussian correlation structure:

rma_gau_Review1_abun_lat<-rma.mv(es_dung$yi ~ abs(lat) + diff_cow.dens + abun, V = es_dung$vi, data = preds_Review1, method = "ML", test = "t", struct = "SPGAU", random = ~ lon + lat|group)

#Rational Quadratic correlation structure:

rma_rat_Review1_abun_lat<-rma.mv(es_dung$yi ~ abs(lat) + diff_cow.dens + abun, V = es_dung$vi, data = preds_Review1, method = "ML", test = "t", struct = "SPRAT", random = ~ lon + lat|group)

#Spherical correlation structure:

rma_sph_Review1_abun_lat<-rma.mv(es_dung$yi ~ abs(lat) + diff_cow.dens + abun, V = es_dung$vi, data = preds_Review1, method = "ML", test = "t", struct = "SPSPH", random = ~ lon + lat|group)

#VIF:

vif(rma_exp_Review1_abun_lat);vif(rma_gau_Review1_abun_lat);

vif(rma_rat_Review1_abun_lat);vif(rma_sph_Review1_abun_lat)

fitstats(rma_exp_Review1_abun_lat)[5];fitstats(rma_gau_Review1_abun_lat)[5];

fitstats(rma_rat_Review1_abun_lat)[5];fitstats(rma_sph_Review1_abun_lat)[5]

rma_exp_Review1_abun_lat #Exponential structure presented the lowest AIC

#Pseudo-R2 following Borenstein et al. 2009:

1-(rma_exp_Review1_abun_lat$tau2/accu_exp$tau2)

#Richness

#Exponential correlation structure:

rma_exp_Review1_rich_lat<-rma.mv(es_dung$yi ~ abs(lat) + diff_cow.dens + rich, V = es_dung$vi, data = preds_Review1, method = "ML", test = "t", struct = "SPEXP", random = ~ lon + lat|group)

#Gaussian correlation structure:

rma_gau_Review1_rich_lat<-rma.mv(es_dung$yi ~ abs(lat) + diff_cow.dens + rich, V = es_dung$vi, data = preds_Review1, method = "ML", test = "t", struct = "SPGAU", random = ~ lon + lat|group)

#Rational Quadratic correlation structure:

```
rma_rat_Review1_rich_lat<-rma.mv(es_dung$yi ~ abs(lat) + diff_cow.dens + rich, V = es_dung$vi, data = preds_Review1, method = "ML", test = "t", struct = "SPRAT", random = ~ lon + lat|group)

#Spherical correlation structure:

rma_sph_Review1_rich_lat<-rma.mv(es_dung$yi ~ abs(lat) + diff_cow.dens + rich, V = es_dung$vi, data = preds_Review1, method = "ML", test = "t", struct = "SPSPH", random = ~ lon + lat|group)

#VIF:

vif(rma_exp_Review1_rich_lat);vif(rma_gau_Review1_rich_lat);

vif(rma_rat_Review1_rich_lat);vif(rma_sph_Review1_rich_lat)

fitstats(rma_exp_Review1_rich_lat)[5];fitstats(rma_gau_Review1_rich_lat)[5];

fitstats(rma_rat_Review1_rich_lat)[5];fitstats(rma_sph_Review1_rich_lat)[5]

rma_exp_Review1_rich_lat #Exponential structure presented the lowest AIC

#Pseudo-R2 following Borenstein et al. 2009:

1-(rma_exp_Review1_rich_lat$tau2/accu_exp$tau2)

#Fdis.behav

#Exponential correlation structure:

rma_exp_Review1_Fdis.behav_lat<-rma.mv(es_dung$yi ~ abs(lat) + diff_cow.dens + Fdis.behav, V = es_dung$vi, data = preds_Review1, method = "ML", test = "t", struct = "SPEXP", random = ~ lon + lat|group)

#Gaussian correlation structure:

rma_gau_Review1_Fdis.behav_lat<-rma.mv(es_dung$yi ~ abs(lat) + diff_cow.dens + Fdis.behav, V = es_dung$vi, data = preds_Review1, method = "ML", test = "t", struct = "SPGAU", random = ~ lon + lat|group)

#Rational Quadratic correlation structure:

rma_rat_Review1_Fdis.behav_lat<-rma.mv(es_dung$yi ~ abs(lat) + diff_cow.dens + Fdis.behav, V = es_dung$vi, data = preds_Review1, method = "ML", test = "t", struct = "SPRAT", random = ~ lon + lat|group)

#Spherical correlation structure:

rma_sph_Review1_Fdis.behav_lat<-rma.mv(es_dung$yi ~ abs(lat) + diff_cow.dens + Fdis.behav, V = es_dung$vi, data = preds_Review1, method = "ML", test = "t", struct = "SPSPH", random = ~ lon + lat|group)

#VIF:

vif(rma_exp_Review1_Fdis.behav_lat);vif(rma_gau_Review1_Fdis.behav_lat);

vif(rma_rat_Review1_Fdis.behav_lat);vif(rma_sph_Review1_Fdis.behav_lat)
```

fitstats(rma_exp_Review1_Fdis.behav_lat)[5];fitstats(rma_gau_Review1_Fdis.behav_lat)[5];

fitstats(rma_rat_Review1_Fdis.behav_lat)[5];fitstats(rma_sph_Review1_Fdis.behav_lat)[5]

rma_exp_Review1_Fdis.behav_lat #Exponential structure presented the #lowest AIC

#Pseudo-R2 following Borenstein et al. 2009:

1-(rma_exp_Review1_Fdis.behav_lat$tau2/accu_exp$tau2)

#Fdis.morph

#Exponential correlation structure:

rma_exp_Review1_Fdis.morph_lat<-rma.mv(es_dung$yi ~ abs(lat) + diff_cow.dens + Fdis.morph, V = es_dung$vi, data = preds_Review1, method = "ML", test = "t", struct = "SPEXP", random = ~ lon + lat|group)

#Gaussian correlation structure:

rma_gau_Review1_Fdis.morph_lat<-rma.mv(es_dung$yi ~ abs(lat) + diff_cow.dens + Fdis.morph, V = es_dung$vi, data = preds_Review1, method = "ML", test = "t", struct = "SPGAU", random = ~ lon + lat|group)

#Rational Quadratic correlation structure:

rma_rat_Review1_Fdis.morph_lat<-rma.mv(es_dung$yi ~ abs(lat) + diff_cow.dens + Fdis.morph, V = es_dung$vi, data = preds_Review1, method = "ML", test = "t", struct = "SPRAT", random = ~ lon + lat|group)

#Spherical correlation structure:

rma_sph_Review1_Fdis.morph_lat<-rma.mv(es_dung$yi ~ abs(lat) + diff_cow.dens + Fdis.morph, V = es_dung$vi, data = preds_Review1, method = "ML", test = "t", struct = "SPSPH", random = ~ lon + lat|group)

#VIF:

vif(rma_exp_Review1_Fdis.morph_lat);vif(rma_gau_Review1_Fdis.morph_lat);

vif(rma_rat_Review1_Fdis.morph_lat);vif(rma_sph_Review1_Fdis.morph_lat)

fitstats(rma_exp_Review1_Fdis.morph_lat)[5];fitstats(rma_gau_Review1_Fdis.morph_lat)[5];

fitstats(rma_rat_Review1_Fdis.morph_lat)[5];fitstats(rma_sph_Review1_Fdis.morph_lat)[5]

rma_exp_Review1_Fdis.morph_lat #Exponential structure presented the lowest AIC

#Pseudo-R2 following Borenstein et al. 2009:

1-(rma_exp_Review1_Fdis.morph_lat$tau2/accu_exp$tau2)

#Models with Latitude and Clim_PCA1

#Meta-regression

#Exponential correlation structure:

rma_exp_Review1_ClimLat<-rma.mv(es_dung$yi ~ Clim_PCA1 + diff_cow.dens + diff_Div_PCA1 + abs(lat), V = es_dung$vi, data = preds_Review1, method = "ML", test = "t", struct = "SPEXP", random = ~ lon + lat|group)

#Gaussian correlation structure:

rma_gau_Review1_ClimLat<-rma.mv(es_dung$yi ~ Clim_PCA1 + diff_cow.dens + diff_Div_PCA1 + abs(lat), V = es_dung$vi, data = preds_Review1, method = "ML", test = "t", struct = "SPGAU", random = ~ lon + lat|group)

#Rational Quadratic correlation structure:

rma_rat_Review1_ClimLat<-rma.mv(es_dung$yi ~ Clim_PCA1 + diff_cow.dens + diff_Div_PCA1 + abs(lat), V = es_dung$vi, data = preds_Review1, method = "ML", test = "t", struct = "SPRAT", random = ~ lon + lat|group)

#Spherical correlation structure:

rma_sph_Review1_ClimLat<-rma.mv(es_dung$yi ~ Clim_PCA1 + diff_cow.dens + diff_Div_PCA1 + abs(lat), V = es_dung$vi, data = preds_Review1, method = "ML", test = "t", struct = "SPSPH", random = ~ lon + lat|group)

#VIF:

vif(rma_exp_Review1_ClimLat);vif(rma_gau_Review1_ClimLat);

vif(rma_rat_Review1_ClimLat);vif(rma_sph_Review1_ClimLat)

fitstats(rma_exp_Review1_ClimLat)[5];fitstats(rma_gau_Review1_ClimLat)[5];

fitstats(rma_rat_Review1_ClimLat)[5];fitstats(rma_sph_Review1_ClimLat)[5]

rma_exp_Review1_ClimLat #Exponential structure presented the lowest AIC

#Pseudo-R2 following Borenstein et al. 2009:

1-(rma_exp_Review1_ClimLat$tau2/accu_exp$tau2)

#Meta-regression with each diversity index

#Abundance

#Exponential correlation structure:

rma_exp_Review1_abun_ClimLat<-rma.mv(es_dung$yi ~ Clim_PCA1 + diff_cow.dens + abun + abs(lat), V = es_dung$vi, data = preds_Review1, method = "ML", test = "t", struct = "SPEXP", random = ~ lon + lat|group)

#Gaussian correlation structure:

rma_gau_Review1_abun_ClimLat<-rma.mv(es_dung$yi ~ Clim_PCA1 + diff_cow.dens + abun + abs(lat), V = es_dung$vi, data = preds_Review1, method = "ML", test = "t", struct = "SPGAU", random = ~ lon + lat|group)

#Rational Quadratic correlation structure:

```
rma_rat_Review1_abun_ClimLat<-rma.mv(es_dung$yi ~ Clim_PCA1 + diff_cow.dens + abun + abs(lat), V = es_dung$vi, data = preds_Review1, method = "ML", test = "t", struct = "SPRAT", random = ~ lon + lat|group)

#Spherical correlation structure:

rma_sph_Review1_abun_ClimLat<-rma.mv(es_dung$yi ~ Clim_PCA1 + diff_cow.dens + abun + abs(lat), V = es_dung$vi, data = preds_Review1, method = "ML", test = "t", struct = "SPSPH", random = ~ lon + lat|group)

#VIF:

vif(rma_exp_Review1_abun_ClimLat);vif(rma_gau_Review1_abun_ClimLat);

vif(rma_rat_Review1_abun_ClimLat);vif(rma_sph_Review1_abun_ClimLat)

fitstats(rma_exp_Review1_abun_ClimLat)[5];fitstats(rma_gau_Review1_abun_ClimLat)[5];

fitstats(rma_rat_Review1_abun_ClimLat)[5];fitstats(rma_sph_Review1_abun_ClimLat)[5]

rma_rat_Review1_abun_ClimLat #Exponential structure presented the lowest AIC

#Pseudo-R2 following Borenstein et al. 2009:

1-(rma_rat_Review1_abun_ClimLat$tau2/accu_exp$tau2)

#Richness

#Exponential correlation structure:

rma_exp_Review1_rich_ClimLat<-rma.mv(es_dung$yi ~ Clim_PCA1 + diff_cow.dens + rich + abs(lat), V = es_dung$vi, data = preds_Review1, method = "ML", test = "t", struct = "SPEXP", random = ~ lon + lat|group)

#Gaussian correlation structure:

rma_gau_Review1_rich_ClimLat<-rma.mv(es_dung$yi ~ Clim_PCA1 + diff_cow.dens + rich + abs(lat), V = es_dung$vi, data = preds_Review1, method = "ML", test = "t", struct = "SPGAU", random = ~ lon + lat|group)

#Rational Quadratic correlation structure:

rma_rat_Review1_rich_ClimLat<-rma.mv(es_dung$yi ~ Clim_PCA1 + diff_cow.dens + rich + abs(lat), V = es_dung$vi, data = preds_Review1, method = "ML", test = "t", struct = "SPRAT", random = ~ lon + lat|group)

#Spherical correlation structure:

rma_sph_Review1_rich_ClimLat<-rma.mv(es_dung$yi ~ Clim_PCA1 + diff_cow.dens + rich + abs(lat), V = es_dung$vi, data = preds_Review1, method = "ML", test = "t", struct = "SPSPH", random = ~ lon + lat|group)

#VIF:

vif(rma_exp_Review1_rich_ClimLat);vif(rma_gau_Review1_rich_ClimLat);

vif(rma_rat_Review1_rich_ClimLat);vif(rma_sph_Review1_rich_ClimLat)
```

fitstats(rma_exp_Review1_rich_ClimLat)[5];fitstats(rma_gau_Review1_rich_ClimLat)[5];

fitstats(rma_rat_Review1_rich_ClimLat)[5];fitstats(rma_sph_Review1_rich_ClimLat)[5]

rma_exp_Review1_rich_ClimLat #Exponential structure presented the lowest #AIC

#Pseudo-R2 following Borenstein et al. 2009:

1-(rma_exp_Review1_rich_ClimLat$tau2/accu_exp$tau2)

#Fdis.behav

#Exponential correlation structure:

rma_exp_Review1_Fdis.behav_ClimLat<-rma.mv(es_dung$yi ~ Clim_PCA1 + diff_cow.dens + Fdis.behav + abs(lat), V = es_dung$vi, data = preds_Review1, method = "ML", test = "t", struct = "SPEXP", random = ~ lon + lat|group)

#Gaussian correlation structure:

rma_gau_Review1_Fdis.behav_ClimLat<-rma.mv(es_dung$yi ~ Clim_PCA1 + diff_cow.dens + Fdis.behav + abs(lat), V = es_dung$vi, data = preds_Review1, method = "ML", test = "t", struct = "SPGAU", random = ~ lon + lat|group)

#Rational Quadratic correlation structure:

rma_rat_Review1_Fdis.behav_ClimLat<-rma.mv(es_dung$yi ~ Clim_PCA1 + diff_cow.dens + Fdis.behav + abs(lat), V = es_dung$vi, data = preds_Review1, method = "ML", test = "t", struct = "SPRAT", random = ~ lon + lat|group)

#Spherical correlation structure:

rma_sph_Review1_Fdis.behav_ClimLat<-rma.mv(es_dung$yi ~ Clim_PCA1 + diff_cow.dens + Fdis.behav + abs(lat), V = es_dung$vi, data = preds_Review1, method = "ML", test = "t", struct = "SPSPH", random = ~ lon + lat|group)

#VIF:

vif(rma_exp_Review1_Fdis.behav_ClimLat);vif(rma_gau_Review1_Fdis.behav_ClimLat);

vif(rma_rat_Review1_Fdis.behav_ClimLat);vif(rma_sph_Review1_Fdis.behav_ClimLat)

fitstats(rma_exp_Review1_Fdis.behav_ClimLat)[5];fitstats(rma_gau_Review1_Fdis.behav_ClimLat)[5];

fitstats(rma_rat_Review1_Fdis.behav_ClimLat)[5];fitstats(rma_sph_Review1_Fdis.behav_ClimLat)[5]

rma_exp_Review1_Fdis.behav_ClimLat #Exponential structure presented the lowest AIC

#Pseudo-R2 following Borenstein et al. 2009:

1-(rma_exp_Review1_Fdis.behav_ClimLat$tau2/accu_exp$tau2)

#Fdis.morph

#Exponential correlation structure:

rma_exp_Review1_Fdis.morph_ClimLat<-rma.mv(es_dung$yi ~ Clim_PCA1 + diff_cow.dens + Fdis.morph + abs(lat), V = es_dung$vi, data = preds_Review1, method = "ML", test = "t", struct = "SPEXP", random = ~ lon + lat|group)

#Gaussian correlation structure:

rma_gau_Review1_Fdis.morph_ClimLat<-rma.mv(es_dung$yi ~ Clim_PCA1 + diff_cow.dens + Fdis.morph + abs(lat), V = es_dung$vi, data = preds_Review1, method = "ML", test = "t", struct = "SPGAU", random = ~ lon + lat|group)

#Rational Quadratic correlation structure:

rma_rat_Review1_Fdis.morph_ClimLat<-rma.mv(es_dung$yi ~ Clim_PCA1 + diff_cow.dens + Fdis.morph + abs(lat), V = es_dung$vi, data = preds_Review1, method = "ML", test = "t", struct = "SPRAT", random = ~ lon + lat|group)

#Spherical correlation structure:

rma_sph_Review1_Fdis.morph_ClimLat<-rma.mv(es_dung$yi ~ Clim_PCA1 + diff_cow.dens + Fdis.morph + abs(lat), V = es_dung$vi, data = preds_Review1, method = "ML", test = "t", struct = "SPSPH", random = ~ lon + lat|group)

#VIF:

vif(rma_exp_Review1_Fdis.morph_ClimLat);vif(rma_gau_Review1_Fdis.morph_ClimLat);

vif(rma_rat_Review1_Fdis.morph_ClimLat);vif(rma_sph_Review1_Fdis.morph_ClimLat)

fitstats(rma_exp_Review1_Fdis.morph_ClimLat)[5];fitstats(rma_gau_Review1_Fdis.morph_ClimLat)[5];

fitstats(rma_rat_Review1_Fdis.morph_ClimLat)[5];fitstats(rma_sph_Review1_Fdis.morph_ClimLat)[5]

rma_exp_Review1_Fdis.morph_ClimLat #Exponential structure presented the lowest AIC

#Pseudo-R2 following Borenstein et al. 2009:

1-(rma_exp_Review1_Fdis.morph_ClimLat$tau2/accu_exp$tau2)
