# Supplementary material for: Dung removal increases under higher dung beetle functional diversity regardless of grazing intensification
Source: Nat Commun. 2023 Dec 6;14:8070. doi: 10.1038/s41467-023-43760-8 (PMC10700315; doi:10.1038/s41467-023-43760-8)
Supplement: Supplementary file 3 — Description of Additional Supplementary Files [file 41467_2023_43760_MOESM3_ESM.pdf]

Description of Additional Supplementary Files

Supplementary Data S1. Codes, location and altitude for all 76 study sites (38 landscapes x 2 sites) where the dung beetle surveys and field experiments on dung removal took place.

Supplementary Data S2. Climatic variables used in the analyses, referred to all 76 study sites.

Supplementary Data S3. Results of all replicates of the dung beetle removal experiments.

Supplementary Data S4. Results of the dung beetle surveys. Numbers indicate the individuals of each species captured by each pitfall trap.

Supplementary Data S5. Summary of trait values for all dung beetle species measured, based on the raw data from S6.

Supplementary Data S6. Trait measurements for all dung beetle individuals measured in the study.

Supplementary Data S7. Diversity metrics measured in all 76 study sites.
